# Study protocol: design and rationale for an exploratory phase II randomized controlled trial to determine optimal vitamin D_3_ supplementation strategies for acute fracture healing

**DOI:** 10.1186/s40814-019-0524-4

**Published:** 2019-11-22

**Authors:** Sheila Sprague, Sofia Bzovsky, Daniel Connelly, Lehana Thabane, Jonathan D. Adachi, Gerard P. Slobogean, on behalf of the Vita-Shock Investigators, Sheila Sprague, Sofia Bzovsky, Daniel Connelly, Lehana Thabane, Jonathan D. Adachi, Gerard P. Slobogean, Mohit Bhandari, Michael F. Holick, Nicole Simunovic, Kim Madden, Heather Dwyer, Taryn Scott, Diane Heels-Ansdell, Joshua Rudnicki, Zachary Hannan, Andrew N. Pollak, Robert V. O’Toole, Christopher LeBrun, Jason W. Nascone, Marcus F. Sciadini, Yasmin Degani, Raymond Pensy, Theodore Manson, W. Andrew Eglseder, Lucas Marchand, Andrea Howe, Dimitrius Marinos, Daniel Mascarenhas, George Reahl, Katherine Ordonio, Marckenley Isaac, Ugochukwu Udogwu, Mitchell Baker, Alexandra Mulliken, Haley Demyanovich

**Affiliations:** 1 Division of Orthopaedic Surgery, Department of Surgery, McMaster University, 1280 Main Street West, Hamilton, Ontario L8S 4L8 Canada; 2 Department of Health Research Methods, Evidence, and Impact, McMaster University, 1280 Main Street West, Hamilton, Ontario L8S 4L8 Canada; 3 R Adams Cowley Shock Trauma Center, Department of Orthopaedics, University of Maryland School of Medicine, 22 South Greene Street, Baltimore, MD 21201 USA; 4 Department of Medicine, McMaster University, 1280 Main Street West, Hamilton, Ontario L8S 4L8 Canada

**Keywords:** Clinical protocols, Tibial shaft fractures, Femoral shaft fractures, Fracture fixation, Vitamin D, Randomized controlled trial

## Abstract

**Background:**

Observational studies have found that 75% of healthy adult fracture patients (ages 18–50) have serum 25-hydroxyvitamin D (25(OH)D) levels < 30 ng/mL. Although lower serum 25(OH)D levels have yet to be correlated to fracture healing complications or poor fracture outcomes, many orthopedic surgeons are routinely prescribing vitamin D supplements to improve fracture healing in healthy non-osteoporotic patients. To address this gap in the literature, we propose a phase II exploratory randomized controlled trial comparing three vitamin D_3_ dosing regimens for early surrogate treatment response.

**Methods:**

We will conduct a 4-arm blinded exploratory phase II trial in 96 adults aged 18–50 years with a closed or low-grade open (Gustilo type I or II) tibial or femoral shaft fracture. Eligible patients will be randomized in equal allocation ratio of 1:1:1:1 to one of the treatment groups: (1) 150,000 IU loading dose vitamin D_3_ plus daily dose placebo; (2) loading dose placebo plus 4000 IU vitamin D_3_ per day, (3) loading dose placebo plus 600 IU vitamin D_3_ per day, or (4) loading dose placebo plus daily dose placebo. The primary outcome is fracture healing, assessed as follows: (1) clinical fracture healing measured using the Function IndeX for Trauma, (2) radiographic fracture healing measured using the Radiographic Union Score for Tibial fractures, and (3) biological fracture healing measured using serum levels of cross-linked C-terminal telopeptides of type I collagen and amino-terminal procollagen propeptides of collagen type I. The main secondary outcome will be assessed by measuring serum 25(OH)D levels. All outcome analyses will be exploratory and adhere to the intention-to-treat principle. Per-protocol sensitivity analyses will also be conducted.

**Discussion:**

Study results will be disseminated through a publication in an academic journal and presentations at orthopedic conferences. Study results will inform dose selection for a large definitive randomized controlled trial and provide preliminary clinical data on which dose may improve acute fracture healing outcomes in healthy adult patients (18–50 years) at 3 months.

**Trial registration:**

Vita-Shock (A Blinded Exploratory Randomized Controlled Trial to Determine Optimal Vitamin D_3_ Supplementation Strategies for Acute Fracture Healing) was registered at ClinicalTrials.gov (identifier NCT02786498) prior to enrollment of participants.

## Background

### Profound impact of lower extremity fractures

Numerous negative consequences of lower extremity long bone fractures have been documented, including delayed healing, nonunion, malunion, and significantly delayed functional recovery [1, 2]. Specifically, at 1-year post-injury, most lower extremity long bone fracture patients have not regained their pre-injury function or quality of life [3–6]. Decreasing re-operations, improving fracture healing, and hastening functional recovery remain ongoing priorities for the orthopedic community. To accomplish this, orthopedic surgeons are increasingly using adjuvant medical therapies to complement their surgical interventions.

### Hypovitaminosis D and adult fracture patients

Several observational studies have reported up to 75% of healthy adult fracture patients (ages 18–50) have serum 25-hydroxyvitamin D (25(OH)D) levels < 30 ng/mL [7, 8]. Although these lower serum 25(OH)D levels have yet to be correlated to fracture healing complications, poor fracture outcomes and the high prevalence of hypovitaminosis D have prompted many orthopedic surgeons to routinely prescribe vitamin D supplements to improve fracture healing in healthy non-osteoporotic patients (ages < 50) [9].

### An emerging practice with no consensus

While evidence-based guidelines recommend vitamin D supplements for general bone health and osteoporosis prevention [10], there is very little data to guide surgeons on the best supplementation strategies or doses to improve fracture healing. Our group recently surveyed 397 orthopedic surgeons and found that more than 29 different dosing regimens of vitamin D were being used to promote fracture healing, with doses ranging from 400 international units (IU) daily to loading doses of 600,000 IU [9]. This difference in practice patterns suggests that a high level of clinical uncertainty exists for this vitamin D indication.

### High-dose supplementation

Many orthopedic surgeons prefer high-dose vitamin D supplementation strategies because of the underlying belief that a rapid increase in available vitamin D may be necessary to maximize fracture healing. This rationale is supported by the chronologic steps of bone healing. Lower extremity long bone fractures heal by callus formation, which begins to form within weeks of injury. Experimental studies have implicated vitamin D in this process and demonstrated that supplementation improves fracture healing in animal models [11–13]. Not only do many patients have low 25(OH)D levels, but recent studies have also identified acute drops in levels shortly after a fracture [14, 15]. Thus, current research provides a mechanistic rationale that supplementation may improve fracture healing. Furthermore, since callus formation is a crucial early healing step, the magnitude of supplementation efficacy may be time dependent, with rapid increases from high doses being beneficial.

### Problem to be addressed

If high doses of vitamin D supplementation improve fracture healing outcomes in healthy non-osteoporotic patients, then there is a large opportunity to increase its use. Some surgeons have suggested that supplementation should be given to all fracture patients [9]. While this approach makes intuitive sense, there are several clinical questions that need to be addressed before widespread adoption is promoted or a definitive efficacy randomized controlled trial (RCT) is initiated. These include the following: (1) Does the timing, frequency, or dose of supplements affect fracture healing?, (2) Are serum 25(OH)D levels associated with fracture healing?, and (3) Does the response to supplementation differ based on a patients’ serum 25(OH)D status? If these questions are not addressed, then there may be a missed opportunity (e.g., ineffective dose, duration, or patient selection) to optimize outcomes in adult fracture patients.

### Progression to a definitive randomized controlled trial

Using a phase II exploratory design, we will test three common dosing strategies of vitamin D_3_ for early surrogate treatment response. Given the paucity of literature on the efficacy of vitamin D supplementation and fracture healing, a phase II exploratory trial is the necessary first step in determining the response of vitamin D and the optimal dosing in a fracture patient population. We will use our study results to design a future definitive RCT that will determine if a high dose of vitamin D_3_ provides a greater response than a common low dose (600 IU) or placebo to reduce re-operations in healthy adult fracture patients (ages 18–50). Selecting which high dose to be used in the future trial is the overarching objective of this exploratory study. Through the results of our long-term definitive trial, we will be poised to have an immediate global impact on the care of fracture patients.

### Vita-Shock exploratory trial

We propose a 96-patient, 4-arm blinded exploratory trial that will compare two high-dose regimens (loading dose and daily dose), a low-dose vitamin D_3_ supplementation, and a placebo for fracture healing in non-osteoporotic patients (ages 18–50). The impetus for our study is fueled by our team’s previous research demonstrating the poor outcomes of tibia and femur fractures and the lack of evidence-based guidance for the dose, frequency, or duration of vitamin D supplements to improve fracture healing. Our proposed study will test the central hypothesis that vitamin D_3_ dose and timing of administration is critical for improving fracture healing at 3 months.

## Study aims and objectives

### Primary feasibility objective

The primary aim is to assess the feasibility of vitamin D_3_ supplementation on fracture healing at 3 months. Fracture healing will be assessed as follows: (1) clinical fracture healing will be measured using the Function IndeX for Trauma (FIX-IT) [16], (2) radiographic fracture healing will be measured using the Radiographic Union Score for Tibial fractures (RUST) [17–20], and (3) biological fracture healing will be measured using serum levels of cross-linked C-terminal telopeptides of type I collagen (CTX) and amino-terminal procollagen propeptides of collagen type I (PINP) [21].

### Secondary feasibility objectives

The main secondary aim is to determine if 25(OH)D serum levels are associated with fracture healing at 3 months. The other secondary objective is to confirm study protocol feasibility for a larger definitive RCT to determine the optimal vitamin D_3_ dosing regimen to reduce re-operations for fracture healing complications in healthy adult patients. Feasibility outcomes include rate of participant enrolment (24 months to enroll 96 participants), adherence with the daily and loading dose vitamin D supplementation (at least 80% compliance), compliance with blood draws (at least 80% compliance), proportion of participants with complete follow-up at 3 months and 12 months post-fracture (90% follow-up at 3 months and 80% at 12 months post-fracture), and level of data quality (95% complete data for completed visits).

### Hypotheses for the primary and secondary feasibility objectives

#### Primary feasibility objective

Lower extremity shaft fractures heal via callus formation and secondary bone healing. This seminal process begins within a few weeks of injury and vitamin D metabolites have been extensively implicated in this stage of healing. During these early weeks, circulating vitamin D levels are most likely to be critical to bone healing; therefore, we hypothesize the following:

1. *High doses (loading or daily) will increase healing compared to low daily dose*. Using high doses will rapidly increase the circulating vitamin D available during fracture callus formation.
2. *High loading dose increases healing compared to high daily dose.* Loading doses will overcome medication adherence issues and increase circulating vitamin D even more rapidly than daily doses.
3. *Low daily dose will increase healing compared to placebo*. While the low daily dose is not expected to increase circulating vitamin D as rapidly as the high-dose strategies, this comparison will determine if rapid serum increases are necessary to improve fracture healing.

#### Secondary feasibility objective

Based on experimental data and the role of vitamin D on bone metabolism, a correlation between circulating vitamin D levels and fracture healing is expected [11–15]; however, the potential efficacy of various supplementation strategies may be dependent on the patient’s baseline vitamin D status or other related changes. For example, it is known that the dose response of supplementation varies depending on the patient’s serum 25(OH)D levels, with larger increases seen in patients with serum levels < 20 ng/ml.

## Methods

### Study setting

The Vita-Shock trial will be conducted at the R Adams Cowley Shock Trauma Center (STC) in Baltimore, Maryland, USA, that treats femoral or tibial shaft fractures in young adults. The Vita-Shock trial was registered at ClinicalTrials.gov (identifier NCT02786498) on June 1, 2016 (https://clinicaltrials.gov/ct2/show/NCT02786498). This protocol paper adheres to the SPIRIT checklist (Additional file 1) as a guide for reporting.

### Inclusion criteria

Patients who meet all the following criteria will be included in the study:

1. Adult men or women aged 18–50 years
2. Closed or low-grade open (Gustilo type I or II) tibial or femoral shaft fracture [22]
3. Fracture treated with a reamed, locked, intramedullary nail
4. Acute fracture (enrolled within 7 days of injury)
5. Provision of informed consent

**Fig. 1:**
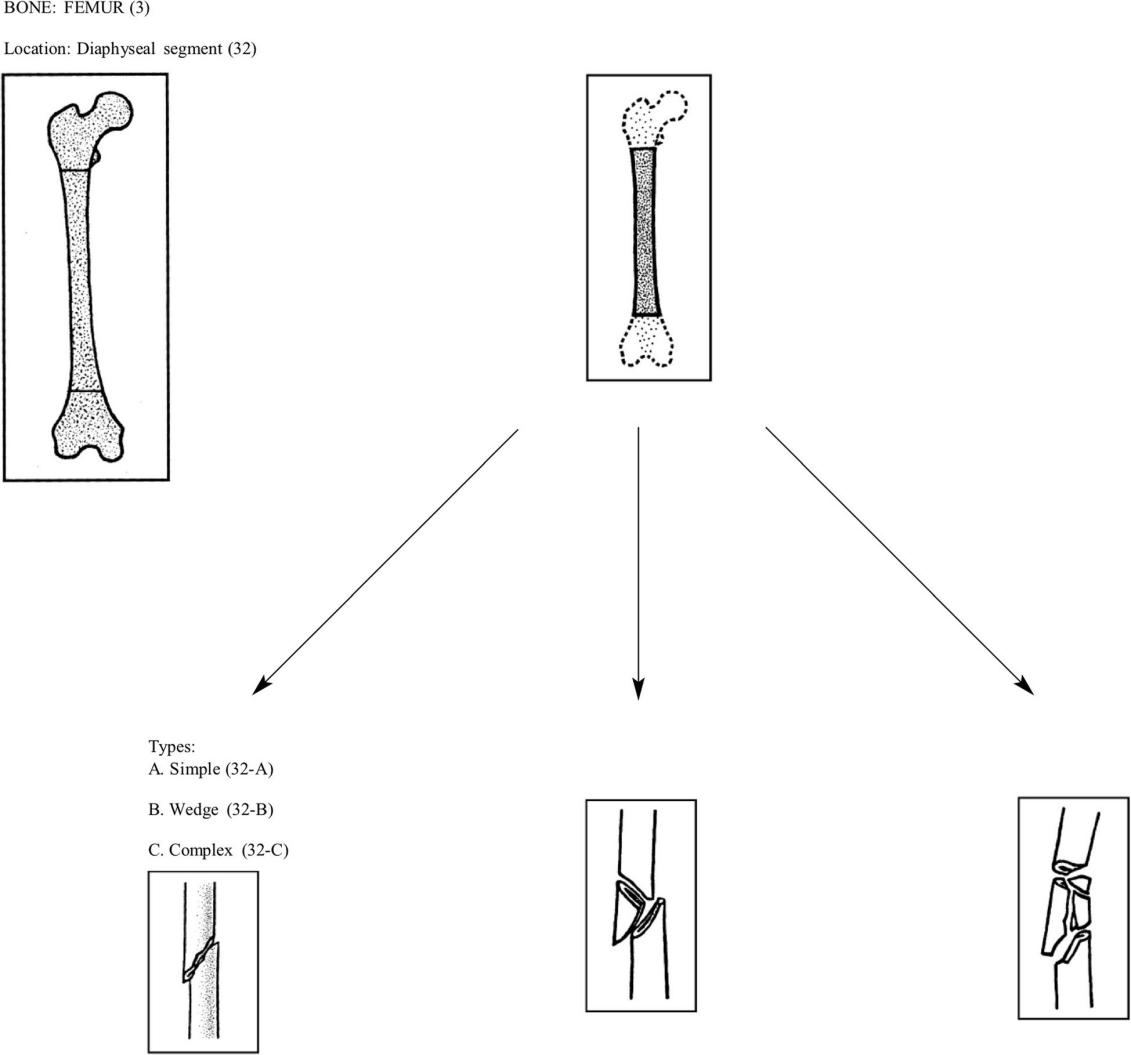
Femur fracture

**Fig. 2:**
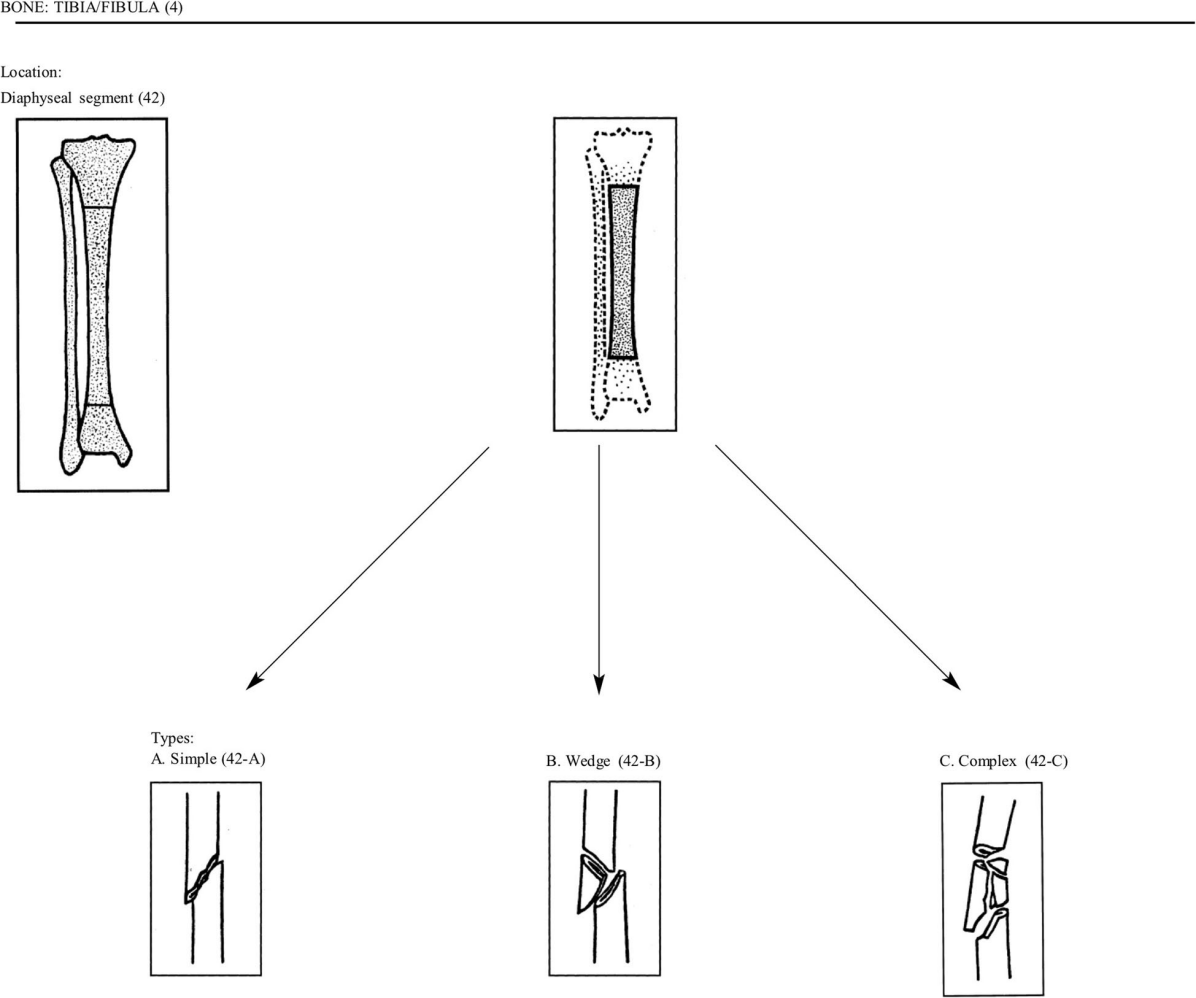
Tibia fracture

Fifty years was selected as the upper age limit to minimize potential confounding with post-menopausal endocrine changes that affect bone metabolism. For the purposes of the study, femoral shaft fractures will be defined as any injury in which the majority of fracture line is distal to the lesser trochanter and proximal to the distal metaphyseal flare of the femoral condyles (Fig. 1). Intertochanteric extension or distal articular extension is permitted. Similarly, a tibial shaft fracture will be defined as an injury with a primary fracture line between the proximal meta-diaphyseal flare to the distal metaphyseal region ending one joint width proximal to the tibial plafond (Fig. 2). Intra-articular extension is permitted.

### Exclusion criteria

Patients who meet any one or more of the following criteria will be excluded from the study:

1. Osteoporosis (a participant was deemed to have osteoporosis if there was prior medical evidence in their medical records or whether the patient indicated that they had this condition. We will not use bone scans to diagnose osteoporosis as part of the study protocol.)
2. Stress fractures
3. Elevated serum calcium (> 10.5 mg/dL)
4. Atypical femur fractures as defined by American Society for Bone and Mineral Research criteria [23]
5. Pathological fractures secondary to neoplasm or other bone lesion
6. Patients with known or likely undiagnosed disorders of bone metabolism such as Paget’s disease, osteomalacia, osteopetrosis, and osteogenesis imperfecta
7. Patients with hyperhomocysteinemia
8. Patients with an allergy to vitamin D or another contraindication to being prescribed vitamin D
9. Patients currently taking an over the counter multivitamin that contains vitamin D and are unable or unwilling to discontinue its use for this study
10. Patients who will likely have problems, in the judgment of the investigators, with maintaining follow-up
11. Pregnancy
12. Patients who are incarcerated
13. Patients who are not expected to survive their injuries
14. Other lower extremity injuries that prevent bilateral full weight-bearing by 6 weeks post-fracture.

Patients with multiple injuries or multiple tibial and femoral shaft fractures will be eligible for inclusion; however, only the most severe eligible fracture will be included (as determined by the treating surgeon using the grade of soft tissue injury using the Tscherne classification system for closed fractures [24] and the Gustilo classification system for open fractures) [22].

### Recruitment strategy and patient screening

All patients presenting to participating surgeons between the ages of 18 to 50 years with a tibial or femoral shaft fracture will be screened. Potentially eligible patients will be approached to participate in the trial. All screened patients will be classified as included or excluded.

### Allocation of patients to study groups

Each participant will be randomized to one of four treatment groups: (1) 150,000 IU loading dose vitamin D_3_ plus daily dose placebo, (2) loading dose placebo plus 4000 IU vitamin D_3_ per day, (3) loading dose placebo plus 600 IU vitamin D_3_ per day, or (4) loading dose placebo plus daily dose placebo. The daily vitamin D_3_ supplements/placebo will be provided in a blinded manner. The daily treatment will commence within 1 week of injury and will be taken for 3 months. The loading dose vitamin D_3_ supplements/placebo will be given within 1 week of injury and at 6 weeks (± 2 weeks) post-injury.

Allocation to the four study groups will be concealed using a centralized 24-h computerized randomization system that will allow internet-based allocation. The treatment allocation will be stratified on the following prognostic factors to ensure balance between the intervention groups: fracture type (closed vs. open) and long bone fracture (tibia vs. femur).

### Vitamin D_3_ (cholecalciferol) treatment groups

#### Blinded administration

The loading dose of 150,000 IU will consist of three 50,000 IU capsules of vitamin D_3_. The loading dose placebo will consist of three capsules that are identical to the 50,000 IU capsules with no active ingredient. The loading dose vitamin D_3_ supplements/placebo will be given within 1 week of injury and at 6 weeks (± 2 weeks) post-injury while in hospital or at the outpatient fracture clinic.

The daily vitamin D_3_ supplements/placebo will be provided in a blinded manner and the daily treatment will commence within 1 week of injury. The daily doses (4000 IU, 600 IU, and placebo) will be identical and will be comprised of one capsule. Patients will be given a bottle of either active vitamin D_3_ or placebo capsules and will be instructed to take one capsule daily for 3 months. The placebo capsules will have no active ingredients and will be identical to the vitamin D capsules. To measure supplementation adherence, participants will be asked to bring their bottles to their follow-up visits. At the 3-month visit, participants will return their bottle to the clinical research coordinator. If the participant does not return the bottle, the clinical research coordinator will provide them with an envelope to return it via mail.

**Fig. 3:**
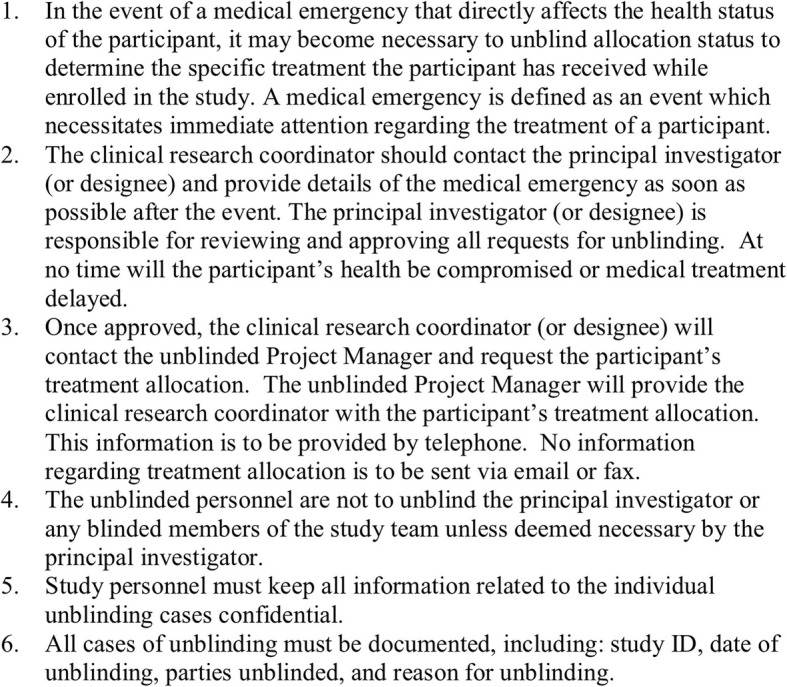
Unblinding of personnel for emergency medical management

All doses of vitamin D and placebo will be obtained from Bio-Tech Pharmacal, Inc. (Fayetteville, AK). The unblinding protocol can be found in Fig. 3. Following the completion of the study, participants may be unblinded their treatment group upon request.

#### Vitamin D_3_ dose rationale

The doses selected are based on biologic rationale, current practice patterns, and existing guidelines. The goal of the high dose arms is to rapidly increase circulating vitamin D and serum 25(OH)D during the early callus fracture healing periods. Conversely, while the low daily dose is not expected to increase circulating vitamin D as quickly as the high-dose strategies, this treatment arm will determine if rapid serum increases are necessary to improve fracture healing. Finally, the placebo control arm is needed to demonstrate the relative potential efficacy of each treatment arm and is also necessary to represent current practice at most trauma centers in North America.

- High loading dose. One hundred fifty thousand international units D_3_ loading doses can be administered easily with three 50,000 IU D_3_ pills. We expect this dose to increase circulating vitamin D levels the fastest. While we acknowledge that many non-orthopedic clinicians may prefer more frequent large doses, such as 50,000 IU weekly, our loading dose strategy has been chosen to correspond with the standard post-operative clinical follow-up schedule. This is important for generalizability and is likely to overcome potential supplementation adherence issues within the adult fracture population that is often predominantly lower socioeconomic patients. This high loading dose is also in the mid-range of other previous large loading doses used safely in fracture patients and is similar to the total cumulative 3-month dose of our high daily dose group.
- High daily dose. Four thousand international units D_3_ represents an alternative high dose strategy and it corresponds to the tolerable upper daily intake level suggested by the Institute of Medicine (IOM) [25]. While this is the IOM’s upper limit, the Endocrine Society has recommended adults can safely take up to 10,000 IU per day [26], further suggesting that our 4000 IU dose should be well-tolerated.
- Low daily dose. Six hundred international units D_3_ is a common dose and approved indication for maintaining general bone health. Six hundred international units is also the IOM’s Recommended Dietary Allowance for all individuals ages 1–70 years [25]. This represents our most conservative supplementation strategy, but its use is common among surgeons prescribing vitamin D and previous studies have shown its efficacy for increasing serum 25(OH)D levels.
- Placebo. Finally, we are including a placebo group because it is important to include placebo-controlled comparisons to our active supplements during this exploratory phase of research. Not only does placebo reflect our usual clinical practice of no supplementation, this exploratory trial will also define our rationale and selection of the control group for the definitive trial. If there are no preliminary response differences between low-dose supplementation and placebo, then the low-dose supplement could be used as the control group in the definitive trial. This would obviate potential criticisms for performing a definitive placebo-controlled trial in a population with a high prevalence of hypovitaminosis D.

#### Storage and administration

As per the standard operating procedures at the STC, the study supplements/placebo will be stored at room temperature in accordance with the manufacturer’s recommendations. The research or pharmacy personnel will maintain an inventory and temperature log to ensure the integrity of the supplements. Study supplementation will begin within 1 week of injury, and it is expected that the research personnel will provide the supplementation to the participant upon discharge from the hospital.

#### Potential adverse events associated with vitamin D

A recent systematic review comprehensively examined the effectiveness and safety of vitamin D supplementation among all ages of adult fracture patients [25]. The majority of research has been performed in elderly fracture populations; however, the safety of a wide range of doses is well established. Studies with doses of 4000 IU daily and loading doses from 50,000 IU for up to 7 days or single loading doses up to 500,000 IU have been used without complication [27].

Since vitamin D regulates parathyroid hormone (PTH) and serum calcium levels, it is theoretically possible that vitamin D supplementation could lead to hypercalcemia. Of the 1088 patients included in the systematic review, four cases of hypercalcemia were reported (0.4%) [28]. Furthermore, there have been no cases of hypercalcemia in several high loading dose clinical trials. Regardless, we will monitor serum calcium levels at enrolment, 6 weeks, and 3 months post-fracture, and clinical signs of hypercalcemia will be sought at all clinical encounters. If hypercalcemia is identified, participants will be instructed to stop their vitamin D supplementation immediately and the hypercalcemia will be treated as indicated.

Finally, we will monitor for increased falls among the study participants. While we do not expect to observe this adverse event in our 18 to 50 year-old adult population, a recent study of 200 elderly fracture patients found that a 60,000 IU monthly loading dose and 24,000 IU monthly loading dose plus 300 μg of calcifediol were associated with increased falls compared to the control group [29]. This single study contradicts several other high loading dose clinical trials, and these concerns have not been borne out of the healthy adult fracture population. This may be a result of the fact that supplementation in the non-osteoporotic fracture population has not been extensively studied (highlighting the need for the proposed research) or because these concerns regarding the risk of falls do not apply to healthy adults without osteoporosis.

#### Concomitant calcium supplementation

In addition, although calcium supplementation is often recommended concomitantly with vitamin D for osteoporosis prevention, for our non-osteoporotic study population the necessity of calcium supplementation is controversial and will not be provided because of the increased risk of kidney stones, hypercalcemia, and potential confounding. This rationale has also been outlined by other researchers performing RCTs involving vitamin D supplementation.

### Surgical technique and post-operative rehabilitation

#### Surgical technique

The study protocol will not dictate the surgical technique. Based on the study’s eligibility criteria, all participants must receive a reamed, locked, intramedullary nail for their tibial or femoral shaft fracture. The number and orientation of locking screws is at the discretion of the treating surgeon, as there have been no studies that demonstrate clinical superiority of any locking screw strategy. Any concomitant fracture lines that extend into the adjacent articular areas may be treated with additional fixation as indicated.

#### Post-operative rehabilitation

Full weight-bearing as tolerated is recommended for all isolated tibial and femoral shaft fractures. In the presence of additional lower extremity fractures, intra-articular extension, or other concomitant soft tissue injuries, participants may be restricted to protected weight-bearing (partial or no weight) for up to 6 weeks post-fracture. If additional contralateral injuries are present, both limbs must be eligible for full weight-bearing by 6 weeks post-fracture.

### Primary and secondary outcome measures

#### Primary outcome

Fracture healing will be assessed as follows: (1) clinical fracture healing will be measured using FIX-IT, (2) radiographic fracture healing will be measured using the RUST, and (3) biological fracture healing will be measured using serum levels of CTX and PINP.

##### Clinical healing

FIX-IT is a standardized measure of weight-bearing and pain in patients with lower extremity fractures, specifically tibia and femur fractures [16]. Preliminary validation of the FIX-IT has demonstrated high inter-rater agreement and moderate correlation with the physical scores of the Short Form-36 [16]. It has been used in other studies to assess clinical fracture healing.

##### Radiographic healing

The RUST score assesses the presence of bridging callus or a persistent fracture line on each of four cortices [17–20]. This score has been previously validated and found to have greater inter-rater reliability when compared with surgeons’ general impression of the cortical bridging [17–20]. RUST has been widely used to assess radiographic fracture healing [17–20]. An orthopedic surgeon who is independent of the study will review the images and assign a RUST score.

##### Biological healing

CTX is a bone-resorption marker and previous research has found that it rises 1 week after fracture of the tibial shaft and remains elevated throughout fracture healing [21]. PINP is a bone-formation marker and prior research has found that it is highest at 12 weeks after fractures of the tibial shaft and proximal femur [30].

The primary time point for assessing fracture healing will be at 3 months post-injury. This time point was selected because it coincides within the standard clinical follow-up schedule, and because it has the greatest potential to detect differences in short-term fracture healing. Additionally, radiographic fracture healing is a surrogate predictive of re-operations related to fracture healing complications. More specifically, the presence of early radiographic callus (< 4 months) has shown to be the strongest predictor of reoperation, with one study reporting 99% accuracy and an area under the curve of 0.995 (*p* < 0.0001) [31]. Data from our institution confirmed these findings with the 3-month RUST score being the most powerful predictor of nonunion surgery for tibia fractures [32]. While we expect the 1-year fracture union rate to be approximately 95% for the femur fractures [30] and 75% for the tibia fractures (unpublished data from the SPRINT trial) [33], improved early fracture healing is biologically plausible. The median time to fracture union for tibia fractures is 4 months; therefore, many patients are still experiencing morbidity from their injury at the 3-month visit and decreasing the time to union would be an important patient benefit.

#### Secondary outcomes

The main secondary outcome for the definitive trial will be assessed by measuring 25(OH)D serum levels. Serum levels will be collected in a blinded manner. Correlations will be assessed between participants’ 25(OH)D levels at enrolment, changes in 25(OH)D levels from enrolment to 3 months, and 25(OH)D levels at 3 months and fracture healing as described above.

The other secondary outcomes will include assessing supplementation adherence between daily and loading doses, confirming participant safety as measured by adverse events and serum levels of calcium and PTH, and assessing protocol adherence (e.g., completion of outcome assessment and participant follow-up).

*Adherence with vitamin D supplementation* will be assessed by participant self-report, by counting the tablets for the daily doses at each follow-up, and by direct observation for the loading doses.

*Participant safety* will be assessed by adverse events, defined as any symptom, sign, illness, or experience that develops or worsens in severity during the course of this study. Within the adverse events collected, fracture healing complications will be identified and will include nonunion (defined as failure of the fracture to progress towards healing for 2 consecutive months and at least 6 months post-fracture), delayed union (defined as a failure of progression of fracture healing beyond the expected median healing time of 4 months with pain at the fracture site), hardware failure (defined as a broken or bent nail or locking screw) [34], wound healing problems (previously published criteria by Anglen [34]), and infection (superficial and deep as defined by Centers for Disease Control and Prevention criteria). Wound healing problems and infection are a part of the composite fracture healing complication outcome because previous animal and infectious disease clinical research has suggested that vitamin D can improve wound healing and reduce infections [35–38]. In addition to adverse events, serum levels of calcium and PTH will be monitored and we will record results of the participants’ pre-operative metabolic profile. These data will be used to understand the baseline metabolic health of the participants and will be used as needed in the event of suspected adverse events.

*Participant adherence with the protocol* will be assessed by monitoring the completion of outcome measures, including clinic assessments (FIX-IT), radiographs (RUST), and blood work (CTX, PINP, 25(OH)D, calcium, and PTH), documentation of adverse events and re-operations, and completion of follow-up to 12 months. Research personnel conducting the outcome assessments will be blinded to the allocation.

### Data collection and participant follow-up

Upon providing informed consent, baseline demographics will be collected from the patient and from their medical chart (Table 1). This includes demographic, medical history, pre-operative blood work-up details (e.g., kidney and liver function tests, calcium, phosphate, and albumin), injury details, fracture characteristics, details on the surgical management of their fracture, and rehabilitation details. Participants will have blood drawn within the fracture clinic that will be analyzed for calcium levels and for CTX, PINP, 25(OH)D, and PTH. Post-operative x-rays will be taken as per standard of care.

**Table 1:** Schedule of events

Assessment	Visit 1: screening and baseline	Visit 2: 6 weeks	Visit 3: 3 months	Visit 4: 6 months	Visit 5: 9 months	Visit 6: 12 months
Screening	●					
Serum calcium analysis	●*	●	●			
Informed consent	●					
Randomization	●					
Collection of baseline data (demographic, serum metabolic panel, fracture, and surgical data)	●					
Nutritional/placebo supplementation**	●	●	●			
Assessment of clinical fracture healing (FIX-IT)		●	●	●	●	●
X-rays of tibia or femur	●	●	●	●	●	●
Assessment of radiographic fracture healing (RUST)		●	●	●	●	●
Serum bone marker analysis (CTX and PINP)	●	●	●			
Assessment of adherence to supplementation		●	●			
Laboratory serum 25(OH)D analysis	●	●	●			
Assessment for adverse events		●	●	●	●	●
Serum PTH level analysis	●	●	●			
Assessment of fracture healing complications		●	●	●	●	●

*To be assessed as eligibility criteria

**Must occur within 1 week of fracture

Feasibility of the study will be assessed over 12 months post-fracture. Participants will be followed at standard clinical visit intervals for 12 months post-injury including 6 weeks, 3 months, 6 months, 9 months, and 12 months. The schedule of events (Table 1) details the requirements and procedures for each visit. Participants will have blood drawn within the fracture clinic that will be analyzed for calcium, CTX, PINP, 25(OH)D, and PTH serum levels at 6 weeks and 3 months. Post-operative x-rays will be taken as per standard of care at each follow-up visit. Participants will be assessed clinically for FIX-IT at each visit. All study outcomes (as defined above) will be documented on the case report forms (CRFs) at each follow-up visit. A 12-month follow-up was selected because it is a standard follow-up period for patients with tibial and femoral shaft fractures and it is a commonly used follow-up period for similar fracture trials [33, 39]. In addition, it is a commonly referenced time period for fracture healing complications requiring reoperation and will further inform decisions surrounding the larger, definitive RCT.

#### Analysis of blood samples

Serum calcium testing will be performed by the hospital laboratory and will be part of the unblinded medical chart for patient safety. The remainder of serum samples (PTH, 25(OH)D, PINP, CTX) will be analyzed in a blinded manner at the end of the study. Laboratory personnel at the University of Maryland’s Muscle Research Laboratory will process the samples from STC for storage in the − 80 °C freezer. Upon completion of all blood work for the study, the serum samples will be transferred to the Institute for Clinical and Translational Research Clinical Research Unit Core Laboratory to be analyzed as a single batch to eliminate inter-batch assay variability. The results of the analyses will be sent to the Center for Evidence-Based Orthopaedics (CEO) to be added to the study database and included within the final data analysis. The treating surgeon will remain blinded to these results. Participants may request the results of their blood analysis at the end of the study.

#### Analysis of radiographs

The radiographs of participants recruited at STC will be stored in the STC Picture Archiving and Communication System and then sent to the CEO for the review of RUST by an independent practicing orthopedic surgeon.

### Participant retention

**Fig. 4:**
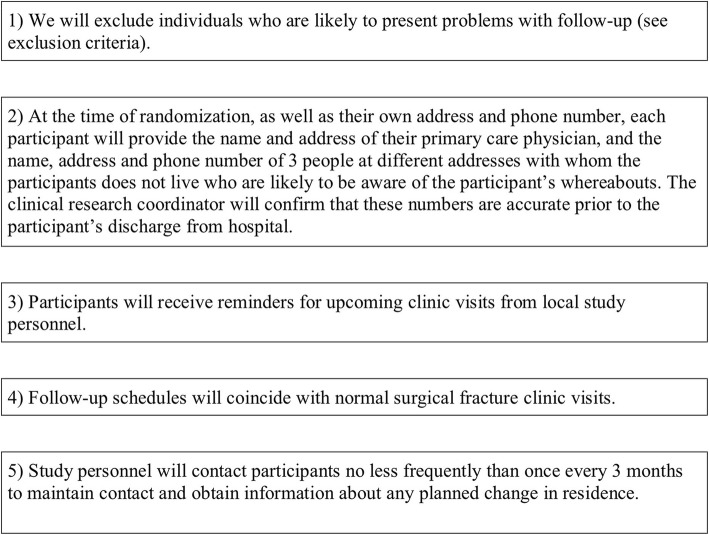
Retention strategies

Once a participant is enrolled in the trial, every reasonable effort will be made to follow the participant for the entire duration of the study period. The expected follow-up rate for this study is greater than 90% based on similar fracture trials [33, 39–41]. To maximize participant retention, all possible attempts should be made to collect as much data as possible and to reduce loss to follow-up. We have implemented procedures to improve participant retention (Fig. 4) [42].

We will only deem participants lost to follow-up after all exhaustive measures have been taken to locate the participant. Participants should not be deemed lost to follow-up until the 12-month visit is due and all attempts to contact the participant have been exhausted. We will not remove participants from the study if the study protocol was not adhered to (e.g., participant received wrong treatment arm, early discontinuation of supplements, occurrence of protocol deviations, missed follow-up visits). We will document the reasons for participant withdrawal from the trial (e.g., withdrawal of consent or lost to follow-up).

## Statistical plan

### Sample size determination

The trial will use a phase II randomized screening design to facilitate non-definitive comparisons of three vitamin D_3_ dosing regimens. Using the principles outlined by Rubinstein et al., the statistical parameters have been carefully chosen to ensure a reasonable sample size for our definitive trial and meaningful results [43]. Consistent with previous recommendations, an *α* and *β* of 0.20 was chosen with a target mean difference of 17–20%, depending on the fracture healing measure. There will be no adjustments for multiple testing given the exploratory nature of the study design.

Based on the original instrument development and validation in tibia and femur fracture patients, it is expected that the low dose and control groups will have a mean 3-month FIX-IT score of 8 (standard deviation (SD) 3) [16]. Assuming the high dose groups will achieve a mean 2-point increase (17% mean difference), 21 patients are required in each group. The same sample size requirements will be applied for comparisons using the RUST instrument based on similar assumptions and recent literature (2-point mean difference, 8 vs. 6, SD 3) [17–20]. Clinically important changes in the PINP and CTX markers are unknown; however, in a previous study of tibia fracture healing, Veitch et al. observed concentrations of both bone turnover markers approximately 100% greater than baseline values [44]. Given the large changes observed in these bone turnover markers, the same criteria will be applied for identifying a potentially clinically beneficial regimen and remain powered to detect a mean difference of 20% (SD 30%). Finally, the sample size will be increased to account for a 10% loss to follow-up, for a total enrolment of 24 patients per allocation group (96 total).

### Statistical methods

All outcome analyses will be exploratory and adhere to the intention-to-treat principle. Per-protocol sensitivity analyses will also be conducted. Our statistical analysis plan will provide additional details on the analyses and will be finalized prior to conducting the final analyses.

#### Specific aim

Each measure of fracture healing will be described with its mean and SD. For our primary analysis, comparisons for the three hypotheses will be made using an independent *t-* test and significance set at *α* = 0.20 (Table 2). Hypothesis 1 compares high-dose supplementation versus low dose. To test this hypothesis, we will combine the two high-dose groups (loading and daily) for a 2:1 comparison against the low daily dose group. All other comparisons will be 1:1 based on the treatment groups outlined.

**Table 2:** Primary outcome analysis

Objective	Hypothesis	Fracture healing outcome	Method of analysis
To determine the response of vitamin D_3_ dose on fracture healing at 3 months	*High doses of supplementation (loading or daily) will increase healing compared to low daily dose*. Using high doses will rapidly increase the circulating vitamin D available during fracture callus formation.	1. FIX-IT (clinical)	Patients in the high loading dose and high daily dose groups will be combined for a 2:1 comparison against low daily dose group using an independent *t*-test (alpha = 0.20).*
		2. RUST (radiographic)	
		3. PINP (biologic)	
		4. CTX (biologic)	
To determine the response of vitamin D_3_ frequency on fracture healing at 3 months	*High loading dose increases healing compared to high daily dose.* Loading doses will overcome medication adherence issues and increase circulating vitamin D even more rapidly than daily doses.	1. FIX-IT (clinical)	Comparisons between the high loading dose and high daily dose groups will be made using an independent t-test (alpha = 0.20).*
		2. RUST (radiographic)	
		3. PINP (biologic)	
		4. CTX (biologic)	
To determine the response of low amounts of vitamin D_3_ supplementation on fracture healing at 3 months	*Low daily dose will increase healing compared to placebo*. While the low daily dose is not expected to increase circulating vitamin D as rapidly as the high dose strategies, this comparison will determine if rapid serum increases are necessary to improve fracture healing.	1. FIX-IT (clinical)	Comparisons between the low daily dose and placebo groups will be made using an independent *t*-test (alpha = 0.20).*
		2. RUST (radiographic)	
		3. PINP (biologic)	
		4. CTX (biologic)	

*Using a phase II screening trial approach, comparisons are non-definitive and an increased alpha level has been adopted

#### Secondary aims

To test the hypotheses of the main secondary aim, adjusted regression models will be used to explore associations between 3-month fracture healing and three assessments of serum 25(OH)D levels: enrolment, 3 months, and change in levels between enrolment and 3 months. Significance will be set at *α* = 0.20. Additional descriptive analyses will be performed for serum 25(OH)D at each time point (Table 3).

**Table 3:** Secondary outcomes analysis

Objective	Hypothesis	Outcome	Method of analysis
Main secondary outcome			
To determine if 25(OH)D serum levels are associated with fracture healing at 3 months	There will be an association between fracture healing and:	1. FIX-IT (Clinical)	Associations will be quantified using univariate analysis (alpha = 0.20).*.
		2. RUST (Radiographic)	
	1) Patients’ enrolment serum 25(OH)D		
		3. PINP (Biologic)	
		4. CTX (Biologic)	
	2) Their change in 25(OH)D from enrolment to 3 months		
	3) Their 25(OH)D level at 3 months		
Other secondary outcomes			
Supplementation adherence	Daily vitamin D_3_ adherence will be < 80% and loading dose vitamin D_3_ adherence will be > 95%.	Self-report	Summary statistics of means and confidence interval.
		Count of pills	
Participant safety	Adverse events will be rare across all 4 treatment groups.	Adverse event	Summary statistics of proportions.
	Re-operations for a composite of fracture healing complications will follow the same 3 hypotheses as fracture healing.	Re-operations for a composite of fracture healing complications	Summary statistics of proportions.
	Levels of serum calcium will be similar across the 4 treatment groups. Levels of serum calcium will be within normal reference ranges.	Serum calcium	Summary statistics of means and confidence interval.
	Levels of serum PTH will be similar across the 4 treatment groups. Levels of serum PTH will be within normal reference ranges.	Serum PTH	Summary statistics of means and confidence interval.
Protocol adherence	Protocol adherence will be acceptable.	Complete follow-up assessments including x-rays and bloodwork	Summary statistics of proportions.

*Using a phase II screening trial approach, comparisons are non-definitive and an increased alpha level has been adopted

All other secondary outcomes will be presented using point estimates and appropriate measures of variance to describe supplementation adherence, participant safety, and key aspects of participant compliance with the protocol (Table 3). Supplement adherence will be summarized using means and 95% confidence intervals (CIs) for participant self-reporting and the mean cumulative dose taken at 3 months. The incidence of adverse events and re-operations for fracture healing complications in each group will be described with counts and proportions. Serum levels of calcium and PTH will be summarized using means and 95% CIs. Participant compliance with the protocol will be summarized descriptively with counts and proportions.

#### Per-protocol sensitivity analyses

The specific aim and the relevant other secondary outcome analyses will be repeated following as-treated analyses. These sensitivity analyses will be completed after the above outcome analyses have been completed and once unblinding has occurred. Per protocol will be defined as participants who received both loading doses of vitamin D and participants who did not miss 20 or more daily doses of vitamin D. Therefore, participants who missed a loading dose of vitamin D and participants who missed 20 or more daily doses of vitamin D will not be included in the as-treated sensitivity analyses.

## Data management

The CRFs will be the primary data collection tool for the study. All data requested on the CRF must be recorded. All data will be entered into the trial database (McMaster University) and double verified.

## Data and safety committee

An orthopedic surgeon at the University of Maryland will monitor patient safety for the Vita-Shock study. The surgeon will be responsible for reviewing adverse events, enrolment numbers, and medical compliance.

All adverse events, both serious and non-serious, will be reported through safety reports that will be sent to the surgeon on an annual basis for review.

Reports will contain the following information:

- Brief narrative introduction that describes the status of the study, progress or findings to-date, issues, and the procedures that produced the report (e.g., data obtained by a specific date).
- Administrative tables that describe study status.
- Aggregate tables of adverse events and serious adverse events.
- Listings of serious adverse events.

### Adverse event reporting and definitions

#### Adverse event

An adverse event is any symptom, sign, illness, or experience that develops or worsens in severity during the course of this study.

#### Serious adverse event

Adverse events are classified as serious or non-serious. A serious adverse event is any adverse event that is any of the following:

- Fatal
- Life threatening
- Requires or prolongs hospital stay
- Results in persistent or significant disability or incapacity
- A congenital anomaly or birth defect
- An important medical event

All serious adverse events must be recorded and promptly submitted to the University of Maryland Institutional Review Board (IRB), as well as be reported to the Methods Centre immediately.

#### Unanticipated problems resulting in risk to participant or others

Any incident, experience, or outcome that meets all of the following criteria:

- Unexpected in nature, severity, or frequency (e.g., not described in study-related documents such as the ethics-approved protocol or consent form).
- Related or possibility related to participation in the research (i.e., possibly related means there is reasonable possibility that the incident experience or outcome may have been caused by the procedures involved in the research).
- Suggests that the research places participants or others at greater risk of harm (including physical, psychological, economic, or social harm).

All unanticipated problems resulting in risk to participants or others must be recorded and promptly submitted to the University of Maryland IRB, as well as be reported to the Methods Centre immediately.

#### Adverse drug reactions

An adverse drug reaction is an injury caused by taking a medication. All adverse drug reactions that are considered both serious and unexpected are to be reported to the US Food and Drug Administration.

## Dissemination

Results from the study will be disseminated through a publication in an academic journal and through presentations at relevant orthopedic conferences regardless of whether or not there are significant findings. Every attempt will be made to ensure that the amount of time between completion of data collection and release of study findings is minimized.

## Supplementary information

**Additional file 1.** SPIRIT 2013 Checklist.

## Data Availability

All data generated in this study will be analyzed upon completion of participant follow-up and will be published in a journal article in the future.

## Abbreviations

25(OH)D: 25-Hydroxyvitamin D
CEO: Center for Evidence-Based Orthopaedics
CI: Confidence interval
CRF: Case report form
CTX: C-Terminal telopeptides of type I collagen
FIX-IT: Function IndeX for Trauma
IRB: Institutional review board
IU: International units
PINP: Amino-terminal procollagen propeptides of collagen type I
PTH: Parathyroid hormone
RCT: Randomized controlled trial
REB: Research ethics board
RUST: Radiographic union score for tibial fractures
SD: Standard deviation
STC: R Adams Cowley Shock Trauma Center
Vita-Shock: A Blinded Exploratory randomized controlled trial to determine optimal vitamin D3 supplementation strategies for acute fracture healing
